# Case of Nisin Oral Ingestion and its LC-MS/MS Detection in Human Urine Over Time: A Case Report

**DOI:** 10.26502/acmcr.96550707

**Published:** 2025-03-10

**Authors:** Pachiyappan Kamarajan, Ahmad Kassem, Allan Radaic, James Wohlschlegel, Yvonne L. Kapila

**Affiliations:** 1 Department of Biosystems and Function, School of Dentistry, University of California Los Angeles, Los Angeles, California, USA; 2 Department of Biological Chemistry, David Geffen School of Medicine, University of California Los Angeles, Los Angeles, California, USA

**Keywords:** Case Report, Nisin, Urine, Mass spectrophotometry, Antimicrobial peptide, Food preservative

## Abstract

**Objective:**

Nisin is a bioactive peptide with antibacterial properties that has been examined for its therapeutic potential. This case report documents the development of a targeted mass spectrometry assay that accurately measures nisin levels in human urine following oral ingestion.

**Case presentation:**

Nisin ZP was orally ingested by a 59-year-old healthy white female then LC-MS/MS methodology was used to detect its excretion in human urine over time over 2 days. Briefly, 20 grams of nisin were ingested in two doses of 10 grams/150 ml of water. Sterile urine was then collected over 2 days. Urine was centrifuged to precipitate insoluble material followed by filtration through a 10 kilodalton molecular weight cutoff membrane. The filtrate was then concentrated by lyophilization, desalted, and then analyzed by LC-MS/MS. After online fractionation using C18 reversed-phase chromatography, the sample was electrosprayed into a Thermo Fusion Lumos mass spectrometer. Data was acquired using a parallel reaction monitoring (PRM) strategy focused on the +5 charge states of nisin. Extracted ion chromatograms for MS/MS fragments corresponding to those charge states were generated using the skyline algorithm and used for quantitation of nisin. Western blotting was also used to evaluate the presence of nisin in the urine samples.

**Results:**

Orally ingested nisin can be detected early in human urine after oral ingestion. Mass spectrometry data revealed that nisin was detected in urine samples 4–20 hours after the first ingestion and up to 14 hours after the second ingestion, indicating a potentially fast turnover and excretion of nisin in the human body. In line with the mass spectrometry data, immunoblotting data validated the findings, further supporting the notion of a fast turnover and excretion of nisin.

**Conclusion:**

We successfully applied the LC-MS/MS method to analyze nisin in urine obtained after oral administration of therapeutic doses of nisin. To the best of our knowledge, this constitutes the first report of nisin’s detection following human oral ingestion and its presence in urine after excretion.

## Introduction

The natural bacteriocin nisin is a food preservative made by the lactic acid bacteria species *Lactococcus lactis,* commonly added to cheeses, meats, and beverages to slow spoilage caused by Gram-positive bacteria. It has been designated by the FDA as Generally Recognized As Safe (GRAS) [1]. However, numerous studies by us and others have shown that purified nisin also has potent broad-spectrum antimicrobial activity against non-foodborne pathogens while retaining its safety profile in both humans and animals [2–5]. For example, we demonstrated that nisin can interfere with pathogenic oral biofilm structure and function [2,6,7], which is a crucial activity for restoring oral eubiosis, as biofilms contribute to oral disease by protecting the bacteria from environmental stresses, promoting horizontal gene transfer, and altering commensal population structure [8,9]. Nisin reduces oral/periodontal inflammation in dogs and rodents [10,11], directly modulates host immune responses in mice [10,12], and has been used to effectively treat mastitis [13,14], neuroinflammation [15], non-alcoholic fatty liver disease [16], and respiratory tract infections in humans, cows, or rodents [13,14,17]. Further, our study using a mouse model of head and neck squamous cell carcinoma (HNSCC) showed that nisin (95% purity), a highly pure and more water-soluble form of the compound, can reduce tumor burden in mice, extend their lifespan, and be ingested at high concentrations [18]. The study also revealed that nisin decreased HNSCC cell proliferation, orasphere formation, and angiogenic sprouting in a dose-dependent manner.

Mechanistically, we found that nisin significantly and preferentially reduced proliferation and increased apoptosis of HNSCC cells versus that of primary oral keratinocytes and it inhibited HNSCC tumor growth in mice via upregulation of the cation transporter and apoptosis mediator CHAC1 [19]. We further discovered that nisin reduced periodontal pathogen-mediated oral tumor aggressiveness in mice [20]. In addition, it has been reported that nisin also exhibits immunomodulatory activity by decreasing the levels of pro-inflammatory cytokines, such as IL-5 and IL-13, promoting IFN-γ production, and suppressing Th2 cellular immune responses [21,22]. Given that HNSCC is characterized by aberrant inflammasome expression [23–25] and that nisin can selectively downregulate markers of inflammation in multiple human cell types with minimal cytotoxicity [12], together with its demonstrated *in vivo* protection against HNSCC tumor progression, the clinical benefit of nisin as a therapeutic agent against OSCC warrants investigation.

Nisin is being considered as a therapeutic agent given its *in vitro and in vivo* anti-tumorigenic effects. Our previous studies and reviews [18–20,26] highlight that nisin preferentially modulates HNSCC cell proliferation and apoptosis versus primary oral keratinocytes in a dose- and time-dependent manner [19] and significantly reduces tumor burden in an HNSCC mouse model without inducing toxicity [18–20]. Together with the established safety profile of nisin as an FDA-approved food additive, its known biomedical applications [26], its safety in animals [22], its lack of toxicity to different oral cells [2], and its effectiveness against HNSCC cells and tumors, these data support the premise that nisin could be clinically beneficial in the treatment of human oral cancers. Further, in 2 recent case reports of humans with tongue OSCC [27,28], we document that nisin exerts beneficial effects as a therapeutic for oral cancer.

Given this background, we are currently conducting the first-in-human clinical trial of nisin in OSCC patients to establish tolerability and feasibility of the treatment regimen and validate the results of prior animal studies highlighting its anticancer properties (ClinicalTrials.gov; NCT06097468). To this end, we plan to examine the pharmacokinetic properties of nisin by analyzing nisin levels in urine samples from study subjects. The current case report was undertaken to develop an assay for this purpose. Urine was collected from one healthy individual overtime over 2 days following the oral ingestion of a therapeutic dose of nisin, and nisin metabolites were monitored via mass spectrometry analysis to re-confirm the short nisin half-life and determine its rapidly waning excretion in the urine; nisin’s short half-life was previously established in animal serum samples prior to FDA approval [29].

Nisin mediates its action in part by interactions with phospholipid bilayers on cellular membranes [30–34]. Prior pharmacokinetic and pharmacodynamic information indicate that nisin can be absorbed via epithelial tissues [31,34]. Nisin can be absorbed through the vaginal epithelium into the circulation following intravaginal administration in rabbits [29]. Maximum levels of nisin were detected in blood samples after 1 h of treatment. The levels declined to baseline after 12 h, suggesting rapid systemic turnover of nisin. Further, Dreyer et al. [35] reported the migration of nisin across gastrointestinal epithelial and vascular endothelial cells in vitro, showing its potential to cross the gut–blood barrier. Nisin was also partially stable in the harsh conditions of the gastric environment [30]. Thus, nisin can be absorbed through the orodigestive tract but is likely to be rapidly excreted in the urine. This was further evaluated by the current study.

A variety of methods have been used to detect nisin. Most methods of nisin detection have focused on its assessment in culture media using assays, such as agar diffusion [36,37], ELISA methods [38–40], and bioassays [41–43] or by mass spectrometry [44]. There are only a few reports on the detection of nisin in food products, such as cheese and milk, and its detection was via mass spectrometry [45–48]. Specifically, liquid chromatography coupled to mass spectrometry (LC-MS/MS) was used to quantify nisin in food/cheese [45–48]. Ko et al. [48] developed a new LC-MS/MS pretreatment protocol for analysis of nisin A and nisin Z content in cow milk and validated this approach. Although a variety of analytical methods have been used for nisin detection, LC/MS enables an accurate determination of the molecular mass of target molecules in crude samples and is therefore an effective method for the detection of bacteriocins [44,49]. However, to our knowledge, there are no reports on the detection of nisin following its ingestion by humans and its detection in urine following its excretion. Our detection approach was similarly based on LC-MS/MS methodology. Western blotting analysis was also performed on the urine samples to further validate the mass spectrometry findings.

## Case Presentation and Materials and Methods

### Nisin oral administration and urine collection

Nisin ZP (Handary S.A; Brussels, Belgium) was ingested by a 59-year-old healthy white female volunteer. In total, 20 grams of nisin were ingested in two doses of 10 grams/150 ml of water each at noon and 6 pm; which were well tolerated without any adverse events. Sterile urine was then collected at 4 pm (Sample 1) and 8 pm (Sample 2) on the first day of ingestion, and at 6 am (Sample 3), 8 am (Sample 4), 3:30 pm (Sample 5), and 9 pm (Sample 6) the next day. Urine samples were frozen at −80°C immediately upon collection (Table 1).

### Sample preparation protocol for Mass Spectrometry

Six urine samples, including both standard and nisin-spiked samples, were thawed/prepared then centrifuged at 4000×g to pellet large solute materials/proteins. The supernatant from each sample was then filtered using a 10 kDa MWCO Amicon^®^ Ultra Centrifugal Filter to further remove larger protein molecules. The filtered urine was lyophilized under stringent conditions (−50 °C and 0.1 Torr) for 48 hours to ensure complete dryness. The lyophilized cake was resuspended in 0.1% Trifluoroacetic Acid (TFA) for sample homogenization. A subsequent centrifugation step at 12000×g for 5 minutes allowed for the separation of any remaining particulate matter, with the supernatant being carefully transferred into a clean tube. The supernatant underwent desalting using C18 Stage Tips, which were prepared by equilibration in 1% TFA and 50% Acetonitrile (ACN), followed by a washing solution of 5% ACN and 0.1% TFA, and an elution in 40% ACN with 0.1% TFA. The peptides eluted were vacuum dried and then resuspended in 5% Formic Acid (FA).

### Mass Spectrometry

Nisin peptide abundance was measured using liquid chromatography coupled to mass spectrometry (LC-MS/MS). After reconstitution in formic acid, the samples were fractionated online by C18 reversed phase chromatography using a Dionex U3000 HPLC to deliver an increasing gradient of acetonitrile [50]. Peptides were eluted and electrosprayed directly in a Thermofisher Fusion Lumos mass spectrometer. Data were acquired using a parallel reaction monitoring strategy in which the +5 precursor ion of full-length nisin was analyzed by MS/MS [51]. The precursor and fragment ion masses for +5 charge state of nisin utilized for Parallel Reaction Monitoring are shown in Table 2. Extracted ion chromatograms were generated for each fragment ion using the Skyline software package [52]. The area under the curve for each fragment ion was summed to calculate an estimate of nisin abundance.

### Sample preparation for Western blotting

The urine samples were filtered using a 10 kDa MWCO Amicon^®^ ultra centrifugal filter (Cat No: UFC201024, Millipore). The filtrate was used to analyze nisin, while the top retained fraction was used to check for potential nisin-binding proteins that might bind to and shift nisin to a higher molecular weight.

### Western blotting

Western blot analyses were performed on the filtrate and top fractions from the urine samples to detect nisin and nisin binding proteins, respectively. Samples were normalized by equal protein concentration (125 μg) and then mixed with 4X Laemmli sample buffer (Cat. No. 1610747, Bio-Rad) containing β-ME (3:1). The proteins were resolved by 16% Tricine SDS-PAGE (Invitrogen, Cat No. EC6695) then transferred to Immobilon-P membranes. The blots were probed with a custom polyclonal affinity-purified rabbit nisin antibody (proprietary to Kapila lab). The antibody against nisin was produced by co-immunizing rabbits with four short nisin peptide sequences (Pacific Immunology, Ramona, CA). Blots were incubated with horseradish peroxidase-conjugated anti-rabbit antibody (SC-2004, Santa Cruz Biotechnology) and then developed with the ECL-plus detection system (Thermo Fisher Scientific).

## Results

**Figure 1:**
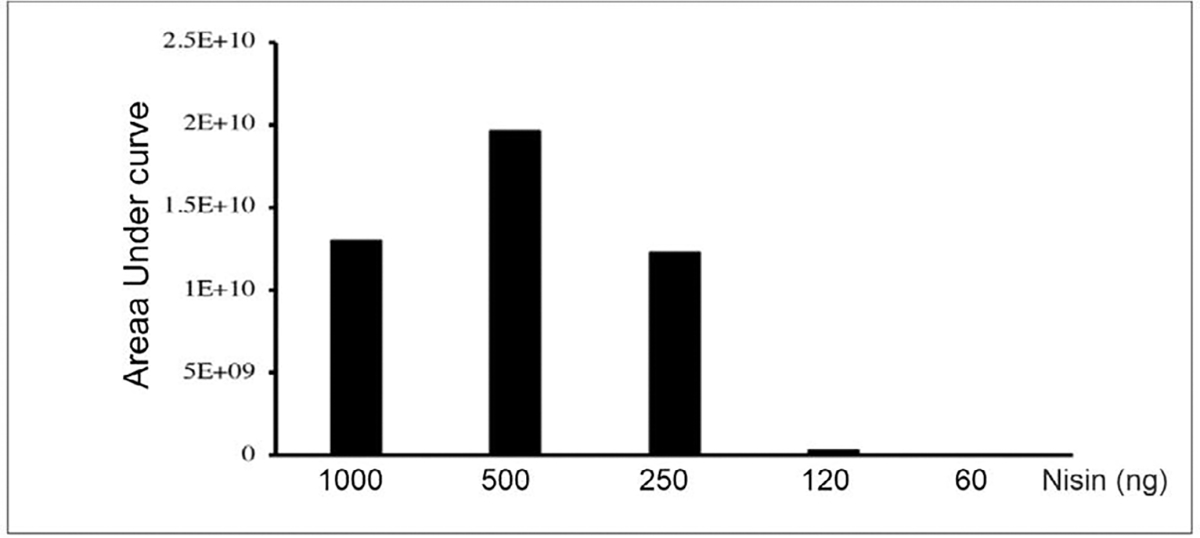
LC-MS/MS analysis of nisin ZP standards. Graph showing nisin abundances in known standards analyzed by LC-MS/MS. The area under the curve for each fragment ion was summed to calculate an estimate of nisin abundance.

**Figure 2:**
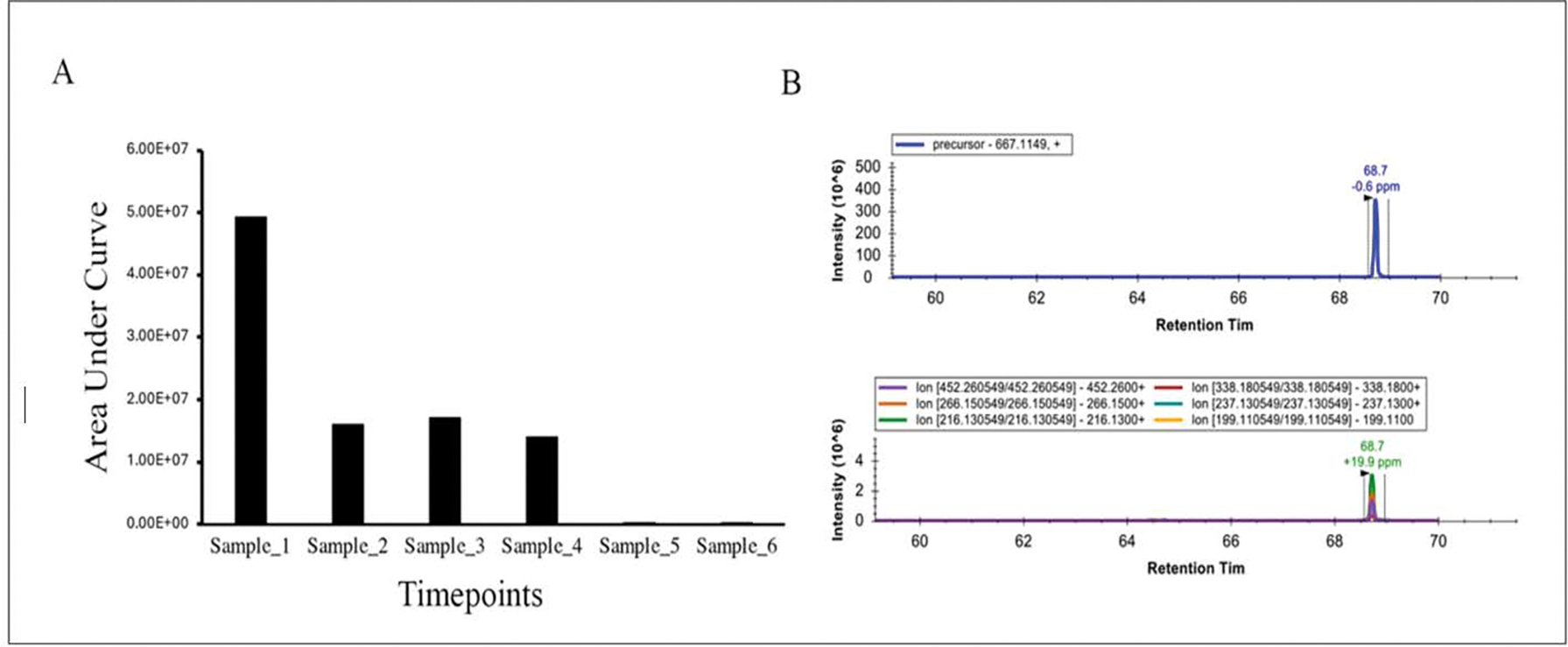
LC-MS/MS Analysis of nisin peptide fragments in urine samples. (A) Graph showing nisin detection in urine samples. (B) Representative extracted ion chromatograms of the intact +5 precursor ion and fragment ions after collision-induced dissociation of the precursor for urine for sample 1.

Nisin abundances are shown in Figure 1 and Figure 2. For Figure 1, peptide abundances were calculated after analyzing known amounts of nisin peptide (60, 120, 250, 500 and 1000 ng) by LC-MS/MS. In this case, nisin was detected at all concentrations but saturated quickly. The limit of detection is likely to be much less than 60 ng and should be explored in future experiments. For Figure 2, nisin was measured in urine samples. Nisin was detected in samples 1–4 but not 5–6. The intact +5 precursor ion chromatograms and fragment ions following collision-induced dissociation of the urine samples and nisin-spiked samples are shown in Supplemental Figures (Figures S1–S10) and additional molecule transition results are shown in the Supplemental Table (Table S1).

**Figure 3:**
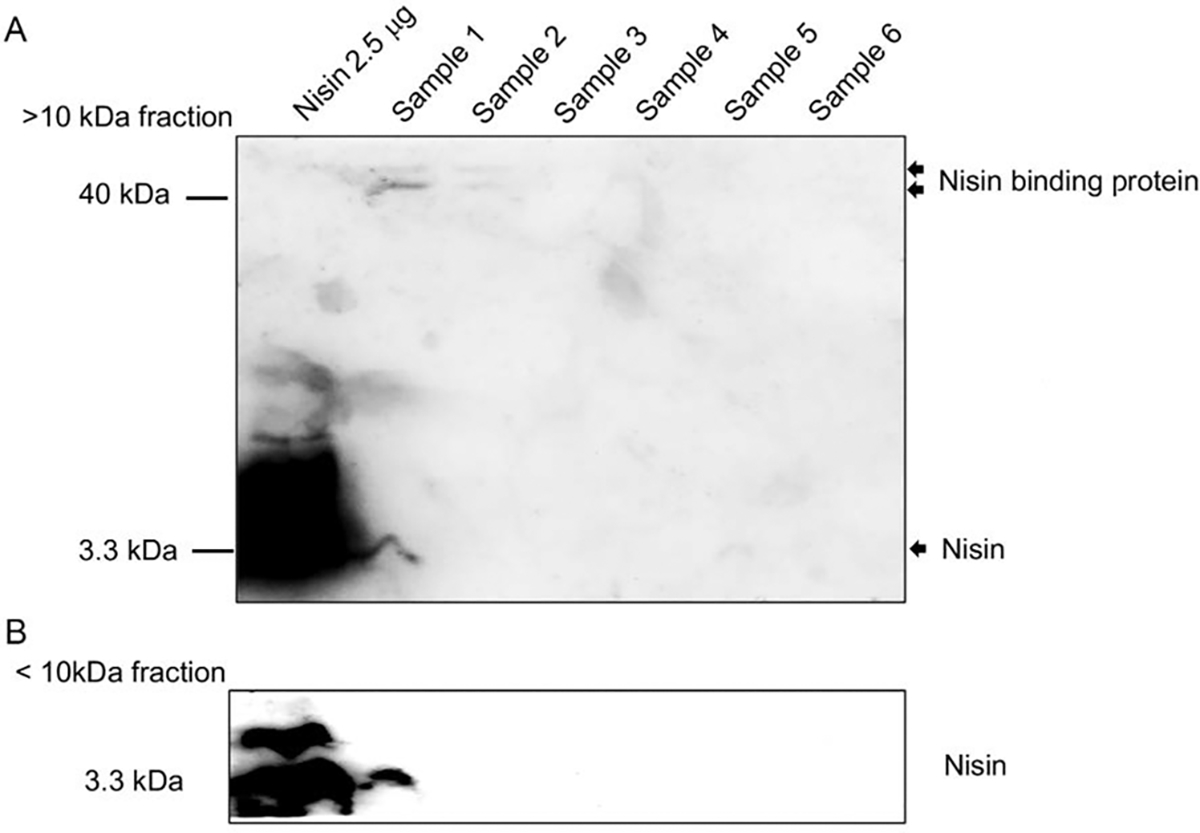
Western blot analysis of nisin in urine samples. The urine samples were filtered using a 10 kDa MWCO Amicon^®^ ultra centrifugal filter. Representative immunoblots show nisin’s presence in >10 kDa fractions (A) and <10 kDa fraction samples (B). Immunoblotting was performed using an anti-nisin antibody. Purified nisin 2.5 mg was loaded onto lane 1 as a positive control, and 125 mg total protein was loaded for each urine sample.

We then further analyzed the presence of nisin in urine samples using immunoblotting with a nisin-specific antibody. Notably, we detected nisin in sample 1 at approximately 3.3 kDa (Figure 3B). We did not detect nisin in samples 2 through 6, thereby showing waning levels of nisin in the urine over time similar to the MS results. Our Western blot results also revealed a high molecular weight nisin-positive band at approximately 40 kDa in sample 1 and to a lesser degree in sample 2, suggesting the presence of a potential nisin-binding protein that likely binds to and shifts nisin to a higher molecular weight position (Figure 3A).

## Discussion

Since nisin was detected in the urine samples in the early time points (Samples 1–4), namely from 4–20 hours after the first ingestion and up to 14 hours after the second ingestion, this indicates that nisin is metabolized and excreted quickly with maximal levels detected as early at 4 hours. Although urine samples from earlier time points prior to 4 hours were not collected, earlier samples might have revealed nisin presence even earlier and at higher levels; potentially showing greater excretion and even faster turnover. Urine sample collection earlier than 4 hours may be beneficial in the future. Furthermore, consistent with the mass spectrometry data, the immunoblotting data also revealed a fast turnover of nisin. This is the first report of nisin detection in human urine following its ingestion and the first report of urine excretion profiles in a human. These data support earlier reports showing that nisin has a fast turnover. Maximum levels of nisin were detected in blood samples after 1 h of treatment/intravaginal administration in rabbits [29]. In rabbits, the levels declined to baseline after 12 hours, suggesting rapid systemic turnover of nisin. Thus, nisin dosing can be absorbed through the orodigestive tract but is rapidly excreted in the urine.

It has been reported that nisin remains intact as it passes through the porcine gastrointestinal tract when ingested orally, as confirmed by mass spectrometry data. This was observed for both encapsulated nisin- and nisin powder-treated groups at 24, 48, and 72 hours [53]. Like other reports of nisin detection with LC-MS/MS, this project employed similar methods to successfully detect nisin and its fragments in urine samples [44–49]. The ongoing clinical trial (ClinicalTrials.gov; NCT06097468) of nisin in oral cancer patients and studies with larger sample sizes will be helpful to further demonstrate nisin’s fast turnover in a unique cohort of oral cancer patients and in healthy cohorts.

## Conclusions

Given that nisin is a commonly used food preservative that has been designated by the FDA as Generally Recognized As Safe (GRAS) since 1988 and has been approved by the Joint Food and Agricultural Organization/World Health Organization as a safe food additive since 1969, plus numerous studies by us and others have shown that nisin has anticancer properties and potent broad-spectrum antimicrobial activity against non-foodborne pathogens while retaining its safety profile in both humans and animals, knowledge about nisin’s pharmacokinetic properties in humans was warranted. In this important case report we document that orally ingested nisin can be detected early in human urine after oral ingestion, indicating a fast turnover and excretion of nisin in the human body. To the best of our knowledge, this constitutes the first report of nisin’s detection following human ingestion and its presence in urine after excretion.

## Supplementary Material

Supplemental Material

## Figures and Tables

**Table 1:** Nisin Z Intake and Urine samples.

Sample No	Nisin intake/Consumption			Urine collection		Hours after ingestion
	Amount	Date	Time	Date	Time	
1	10 g/150 ml	7/17/23	12 noon	7/17/23	4:00 pm	4 hours after first ingestion
2	10 g/150 ml	7/17/23	6:00 pm	7/17/23	8:00 pm	8 hours after first ingestion
						2 hours after second ingestion
3		7/18/23		7/18/23	6:00 am	18 hours after first ingestion
						12 hours after second ingestion
4		7/18/23		7/18/23	8:00 am	20 hours after first ingestion
						14 hours after second ingestion
5		7/18/23		7/18/23	3:30 pm	27.5 hours after first ingestion
						21.5 hours after second ingestion
6		7/18/23		7/18/23	9:00 pm	33 hours after first ingestion
						27 hours after second ingestion

**Table 2:** Precursor and fragment ion masses for +5 charge state of nisin ZP utilized for parallel reaction monitoring.

Precursor Mz	Precursor Charge	Product Mz	Product Charge
667.11493	5	452.26	1
667.11493	5	338.18	1
667.11493	5	266.15	1
667.11493	5	237.13	1
667.11493	5	216.13	1
667.11493	5	199.11	1

## Data Availability

All data and material are provided in the manuscript.
