# Supplementary material for: Case of Nisin Oral Ingestion and its LC-MS/MS Detection in Human Urine Over Time: A Case Report
Source: Arch Clin Med Case Rep. Author manuscript; Available in PMC 2025 Sep 17. (PMC12439769; doi:10.26502/acmcr.96550707)
Supplement: Supplemental Material [file NIHMS2071373-supplement-Supplemental_Material.pdf]

Supplementary Files:

Supplemental Table S1: Molecule Transition Results.

Molecule List Name	Replicate Name	Precursor Mz	Precursor Adduct	Precursor Charge	Fragment Ion	Product Mz	Product Adduct	Product Charge	Retention Time	Area	Background	Peak Rank
molecules1	10ugspiked	667.11493	[M+5]	5	precursor	667.11493	[M+5]	5	65.11	4.53665E+11	5238917120	1
molecules1	20ugspiked	667.11493	[M+5]	5	precursor	667.11493	[M+5]	5	64.74	5.91393E+11	20141570048	1
molecules1	25ugspiked	667.11493	[M+5]	5	precursor	667.11493	[M+5]	5	64.63	6.18731E+11	10158816256	1
molecules1	50ugspiked	667.11493	[M+5]	5	precursor	667.11493	[M+5]	5	63.15	1.06029E+12	34631421952	1
molecules1	100ugspiked	667.11493	[M+5]	5	precursor	667.11493	[M+5]	5	63.5	1.23792E+12	32036059136	1
molecules1	250ugspiked	667.11493	[M+5]	5	precursor	667.11493	[M+5]	5	64.25	7.69301E+11	3751321344	1
molecules1	500ugspiked	667.11493	[M+5]	5	precursor	667.11493	[M+5]	5	69.52	3.78313E+11	15933358	1
molecules1	Sample_1	667.11493	[M+5]	5	precursor	667.11493	[M+5]	5	68.73	1593830400	9584490	1
molecules1	Sample_2	667.11493	[M+5]	5	precursor	667.11493	[M+5]	5	68.81	466066208	8304677	1
molecules1	Sample_3	667.11493	[M+5]	5	precursor	667.11493	[M+5]	5	68.8	568709696	43156132	1
molecules1	Sample_4	667.11493	[M+5]	5	precursor	667.11493	[M+5]	5	68.95	343968384	284106	1
molecules1	Sample_5	667.11493	[M+5]	5	precursor	667.11493	[M+5]	5	21.71	0	0	0
molecules1	Sample_6	667.11493	[M+5]	5	precursor	667.11493	[M+5]	5	41.62	95195	0	1
molecules1	10ugspiked	667.11493	[M+5]	5	Ion [452.260549/452.260549]	452.26	[M+]	1	65.11	645333312	7763673	5
molecules1	20ugspiked	667.11493	[M+5]	5	Ion [452.260549/452.260549]	452.26	[M+]	1	64.7	987145792	46208012	5

molecules1	25ugspiked	667.11493	[M+5]	5	Ion [452.260549/452.260549]	452.26	[M+]	1	65.14	1141044352	32134502	5
molecules1	50ugspiked	667.11493	[M+5]	5	Ion [452.260549/452.260549]	452.26	[M+]	1	63.45	3083715584	208323712	6
molecules1	100ugspiked	667.11493	[M+5]	5	Ion [452.260549/452.260549]	452.26	[M+]	1	63.54	3259003904	160603776	6
molecules1	250ugspiked	667.11493	[M+5]	5	Ion [452.260549/452.260549]	452.26	[M+]	1	64.26	2547777536	9663207	6
molecules1	500ugspiked	667.11493	[M+5]	5	Ion [452.260549/452.260549]	452.26	[M+]	1	69.44	1487766784	3810676	6
molecules1	Sample_1	667.11493	[M+5]	5	Ion [452.260549/452.260549]	452.26	[M+]	1	68.73	6658329	199960	6
molecules1	Sample_2	667.11493	[M+5]	5	Ion [452.260549/452.260549]	452.26	[M+]	1	68.81	2152114	107919	6
molecules1	Sample_3	667.11493	[M+5]	5	Ion [452.260549/452.260549]	452.26	[M+]	1	68.8	2334626	234403	6
molecules1	Sample_4	667.11493	[M+5]	5	Ion [452.260549/452.260549]	452.26	[M+]	1	68.96	1927874	0	6
molecules1	Sample_5	667.11493	[M+5]	5	Ion [452.260549/452.260549]	452.26	[M+]	1	21.71	0	0	0
molecules1	Sample_6	667.11493	[M+5]	5	Ion [452.260549/452.260549]	452.26	[M+]	1	41.59	0	0	0
molecules1	10ugspiked	667.11493	[M+5]	5	Ion [338.180549/338.180549]	338.18	[M+]	1	64.78	153380720	1227397	7
molecules1	20ugspiked	667.11493	[M+5]	5	Ion [338.180549/338.180549]	338.18	[M+]	1	64.7	252044848	8901608	7
molecules1	25ugspiked	667.11493	[M+5]	5	Ion [338.180549/338.180549]	338.18	[M+]	1	64.59	278779968	9822382	7

molecules1	50ugspiked	667.11493	[M+5]	5	Ion [338.180549/338.180549]	338.18	[M+]	1	63.45	903061632	58759408	7
molecules1	100ugspiked	667.11493	[M+5]	5	Ion [338.180549/338.180549]	338.18	[M+]	1	63.53	998730304	37838948	7
molecules1	250ugspiked	667.11493	[M+5]	5	Ion [338.180549/338.180549]	338.18	[M+]	1	64.24	731983808	2170405	7
molecules1	500ugspiked	667.11493	[M+5]	5	Ion [338.180549/338.180549]	338.18	[M+]	1	69.45	408860864	0	7
molecules1	Sample_1	667.11493	[M+5]	5	Ion [338.180549/338.180549]	338.18	[M+]	1	68.73	1751020	0	7
molecules1	Sample_2	667.11493	[M+5]	5	Ion [338.180549/338.180549]	338.18	[M+]	1	68.81	633272	0	7
molecules1	Sample_3	667.11493	[M+5]	5	Ion [338.180549/338.180549]	338.18	[M+]	1	68.82	698470	52677	7
molecules1	Sample_4	667.11493	[M+5]	5	Ion [338.180549/338.180549]	338.18	[M+]	1	68.96	513586	0	7
molecules1	Sample_5	667.11493	[M+5]	5	Ion [338.180549/338.180549]	338.18	[M+]	1	21.71	0	0	0
molecules1	Sample_6	667.11493	[M+5]	5	Ion [338.180549/338.180549]	338.18	[M+]	1	41.62	2151	0	3
molecules1	10ugspiked	667.11493	[M+5]	5	Ion [266.150549/266.150549]	266.15	[M+]	1	64.78	798451520	9279381	3
molecules1	20ugspiked	667.11493	[M+5]	5	Ion [266.150549/266.150549]	266.15	[M+]	1	64.83	1190713088	55779560	3
molecules1	25ugspiked	667.11493	[M+5]	5	Ion [266.150549/266.150549]	266.15	[M+]	1	65.22	1426774912	29985344	3
molecules1	50ugspiked	667.11493	[M+5]	5	Ion [266.150549/266.150549]	266.15	[M+]	1	63.51	4076502272	297507872	3

molecules1	100ugspiked	667.11493	[M+5]	5	Ion [266.150549/266.150549]	266.15	[M+]	1	63.53	4306021888	226063760	3
molecules1	250ugspiked	667.11493	[M+5]	5	Ion [266.150549/266.150549]	266.15	[M+]	1	64.17	3372033536	12915921	3
molecules1	500ugspiked	667.11493	[M+5]	5	Ion [266.150549/266.150549]	266.15	[M+]	1	69.45	1944407680	6028725	3
molecules1	Sample_1	667.11493	[M+5]	5	Ion [266.150549/266.150549]	266.15	[M+]	1	68.73	9277805	188629	3
molecules1	Sample_2	667.11493	[M+5]	5	Ion [266.150549/266.150549]	266.15	[M+]	1	68.81	2911866	113416	3
molecules1	Sample_3	667.11493	[M+5]	5	Ion [266.150549/266.150549]	266.15	[M+]	1	68.81	3010100	365425	3
molecules1	Sample_4	667.11493	[M+5]	5	Ion [266.150549/266.150549]	266.15	[M+]	1	68.96	2656909	50582	3
molecules1	Sample_5	667.11493	[M+5]	5	Ion [266.150549/266.150549]	266.15	[M+]	1	21.71	0	0	0
molecules1	Sample_6	667.11493	[M+5]	5	Ion [266.150549/266.150549]	266.15	[M+]	1	41.59	0	0	0
molecules1	10ugspiked	667.11493	[M+5]	5	Ion [237.130549/237.130549]	237.13	[M+]	1	65.11	586090240	7589477	6
molecules1	20ugspiked	667.11493	[M+5]	5	Ion [237.130549/237.130549]	237.13	[M+]	1	64.71	909351616	35742564	6
molecules1	25ugspiked	667.11493	[M+5]	5	Ion [237.130549/237.130549]	237.13	[M+]	1	65.24	1003177728	32389698	6
molecules1	50ugspiked	667.11493	[M+5]	5	Ion [237.130549/237.130549]	237.13	[M+]	1	63.51	3371748608	217667760	5
molecules1	100ugspiked	667.11493	[M+5]	5	Ion [237.130549/237.130549]	237.13	[M+]	1	63.54	3635740672	148064704	5

molecules1	250ugspiked	667.11493	[M+5]	5	Ion [237.130549/237.130549]	237.13	[M+]	1	64.2	2767659520	9176368	5
molecules1	500ugspiked	667.11493	[M+5]	5	Ion [237.130549/237.130549]	237.13	[M+]	1	69.45	1575986048	4719475	5
molecules1	Sample_1	667.11493	[M+5]	5	Ion [237.130549/237.130549]	237.13	[M+]	1	68.72	7308601	105442	5
molecules1	Sample_2	667.11493	[M+5]	5	Ion [237.130549/237.130549]	237.13	[M+]	1	68.82	2166438	80329	5
molecules1	Sample_3	667.11493	[M+5]	5	Ion [237.130549/237.130549]	237.13	[M+]	1	68.82	2434182	213074	5
molecules1	Sample_4	667.11493	[M+5]	5	Ion [237.130549/237.130549]	237.13	[M+]	1	68.96	2134379	0	5
molecules1	Sample_5	667.11493	[M+5]	5	Ion [237.130549/237.130549]	237.13	[M+]	1	21.71	0	0	0
molecules1	Sample_6	667.11493	[M+5]	5	Ion [237.130549/237.130549]	237.13	[M+]	1	41.59	0	0	0
molecules1	10ugspiked	667.11493	[M+5]	5	Ion [216.130549/216.130549]	216.13	[M+]	1	65.11	1311800576	14539612	2
molecules1	20ugspiked	667.11493	[M+5]	5	Ion [216.130549/216.130549]	216.13	[M+]	1	64.71	1942265600	79803296	2
molecules1	25ugspiked	667.11493	[M+5]	5	Ion [216.130549/216.130549]	216.13	[M+]	1	65.19	2274486784	76861392	2
molecules1	50ugspiked	667.11493	[M+5]	5	Ion [216.130549/216.130549]	216.13	[M+]	1	63.51	6962075136	446268544	2
molecules1	100ugspiked	667.11493	[M+5]	5	Ion [216.130549/216.130549]	216.13	[M+]	1	63.51	7436984320	351468800	2
molecules1	250ugspiked	667.11493	[M+5]	5	Ion [216.130549/216.130549]	216.13	[M+]	1	64.28	5853349376	21668854	2

molecules1	500ugspiked	667.11493	[M+5]	5	Ion [216.130549/216.130549]	216.13	[M+]	1	69.45	3363085568	7860044	2
molecules1	Sample_1	667.11493	[M+5]	5	Ion [216.130549/216.130549]	216.13	[M+]	1	68.73	15346155	415475	2
molecules1	Sample_2	667.11493	[M+5]	5	Ion [216.130549/216.130549]	216.13	[M+]	1	68.81	4945642	238261	2
molecules1	Sample_3	667.11493	[M+5]	5	Ion [216.130549/216.130549]	216.13	[M+]	1	68.82	5428327	652641	2
molecules1	Sample_4	667.11493	[M+5]	5	Ion [216.130549/216.130549]	216.13	[M+]	1	68.96	4515728	51664	2
molecules1	Sample_5	667.11493	[M+5]	5	Ion [216.130549/216.130549]	216.13	[M+]	1	21.8	22701	0	2
molecules1	Sample_6	667.11493	[M+5]	5	Ion [216.130549/216.130549]	216.13	[M+]	1	41.63	3443	0	2
molecules1	10ugspiked	667.11493	[M+5]	5	Ion [199.110549/199.110549]	199.11	[M+]	1	65.11	677471040	10108060	4
molecules1	20ugspiked	667.11493	[M+5]	5	Ion [199.110549/199.110549]	199.11	[M+]	1	64.7	1037787136	41500516	4
molecules1	25ugspiked	667.11493	[M+5]	5	Ion [199.110549/199.110549]	199.11	[M+]	1	64.59	1180375552	41097356	4
molecules1	50ugspiked	667.11493	[M+5]	5	Ion [199.110549/199.110549]	199.11	[M+]	1	64.13	3975561216	264137872	4
molecules1	100ugspiked	667.11493	[M+5]	5	Ion [199.110549/199.110549]	199.11	[M+]	1	63.54	4247725824	192340720	4
molecules1	250ugspiked	667.11493	[M+5]	5	Ion [199.110549/199.110549]	199.11	[M+]	1	64.24	3284340736	13754188	4
molecules1	500ugspiked	667.11493	[M+5]	5	Ion [199.110549/199.110549]	199.11	[M+]	1	69.45	1882955392	4862292	4

molecules1	Sample_1	667.11493	[M+5]	5	Ion [199.110549/199.110549]	199.11	[M+]	1	68.73	8940760	214210	4
molecules1	Sample_2	667.11493	[M+5]	5	Ion [199.110549/199.110549]	199.11	[M+]	1	68.81	2767302	129950	4
molecules1	Sample_3	667.11493	[M+5]	5	Ion [199.110549/199.110549]	199.11	[M+]	1	68.82	2960018	320089	4
molecules1	Sample_4	667.11493	[M+5]	5	Ion [199.110549/199.110549]	199.11	[M+]	1	68.96	2499380	20726	4
molecules1	Sample_5	667.11493	[M+5]	5	Ion [199.110549/199.110549]	199.11	[M+]	1	21.8	39178	0	1
molecules1	Sample_6	667.11493	[M+5]	5	Ion [199.110549/199.110549]	199.11	[M+]	1	41.59	0	0	0

Supplementary Figures:

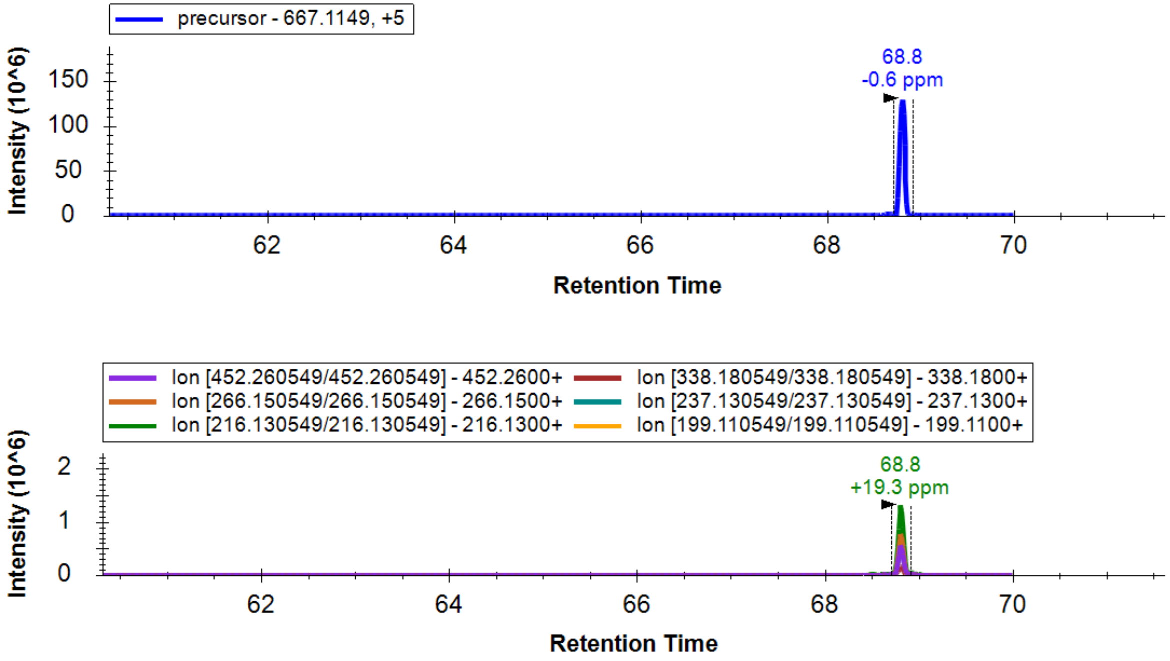

Figure S1: **LC-MS/MS Analysis of nisin peptide in urine.** Nisin was enriched from urine (sample 2) and analyzed by parallel reaction monitoring on a Thermo Fusion Lumos mass spectrometer. Representative extracted ion chromatograms of the intact +5 precursor ion (top) and fragment ions after collision induced dissociation of the precursor (bottom) are shown.

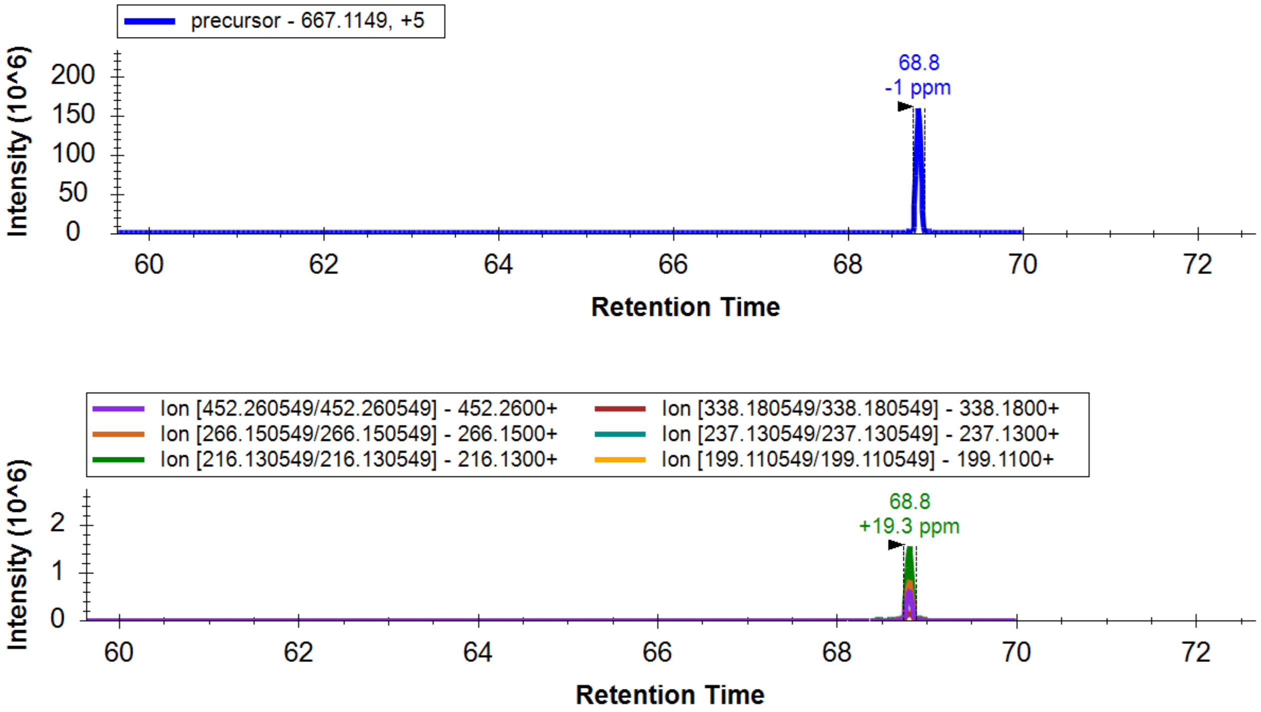

Figure S2: LC-MS/MS Analysis of nisin peptide in urine. Nisin was enriched from urine (sample 3) and analyzed by parallel reaction monitoring on a Thermo Fusion Lumos mass spectrometer. Representative extracted ion chromatograms of the intact +5 precursor ion (top) and fragment ions after collision induced dissociation of the precursor (bottom) are shown.

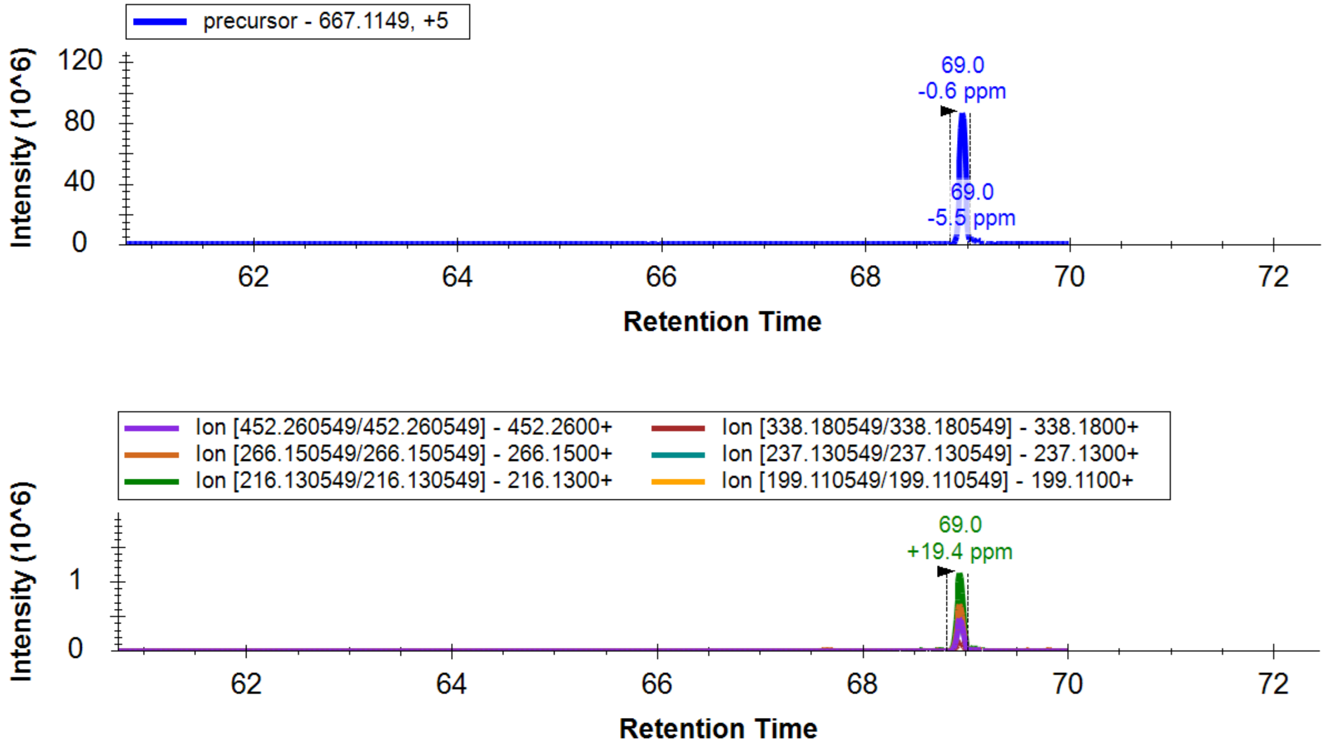

Figure S3: LC-MS/MS Analysis of nisin peptide in urine. Nisin was enriched from urine (sample 4) and analyzed by parallel reaction monitoring on a Thermo Fusion Lumos mass spectrometer. Representative extracted ion chromatograms of the intact +5 precursor ion (top) and fragment ions after collision induced dissociation of the precursor (bottom) are shown.

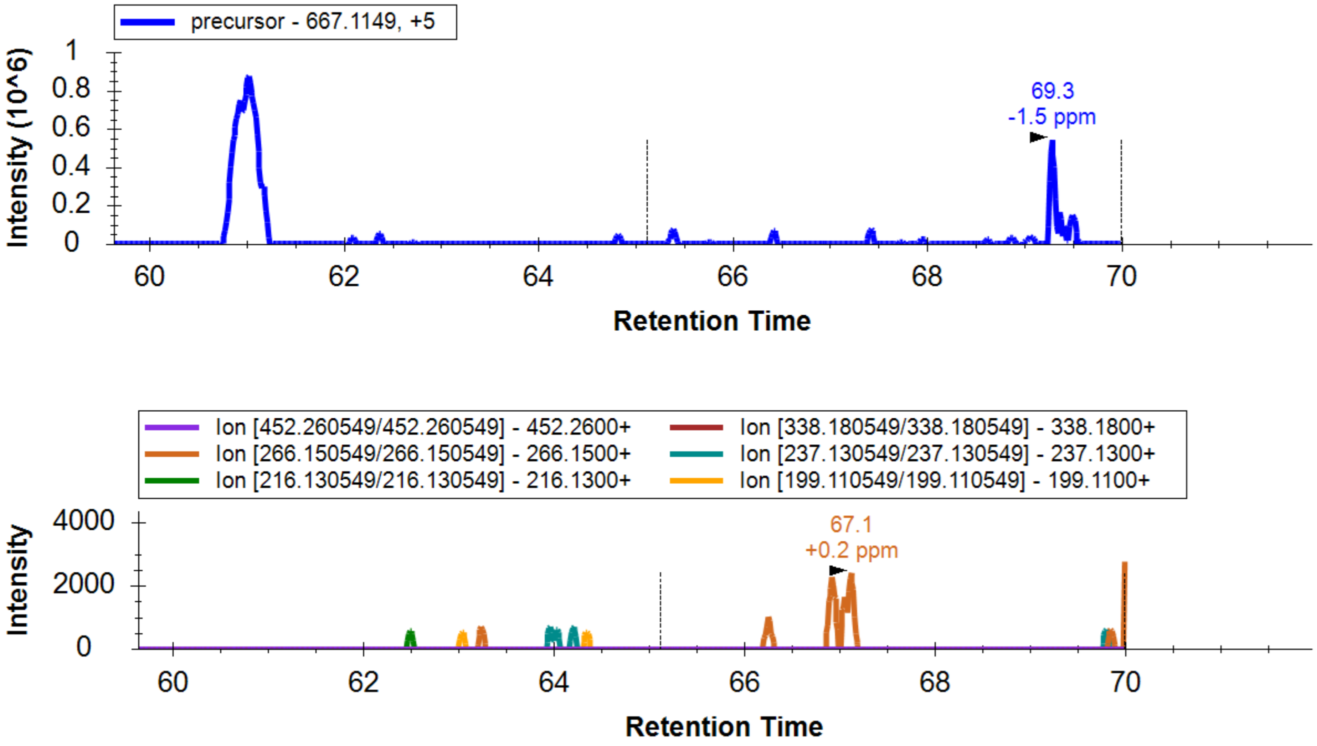

Figure S4: LC-MS/MS Analysis of nisin peptide in urine. Nisin was enriched from urine (sample 5) and analyzed by parallel reaction monitoring on a Thermo Fusion Lumos mass spectrometer. Representative extracted ion chromatograms of the intact +5 precursor ion (top) and fragment ions after collision induced dissociation of the precursor (bottom) are shown.

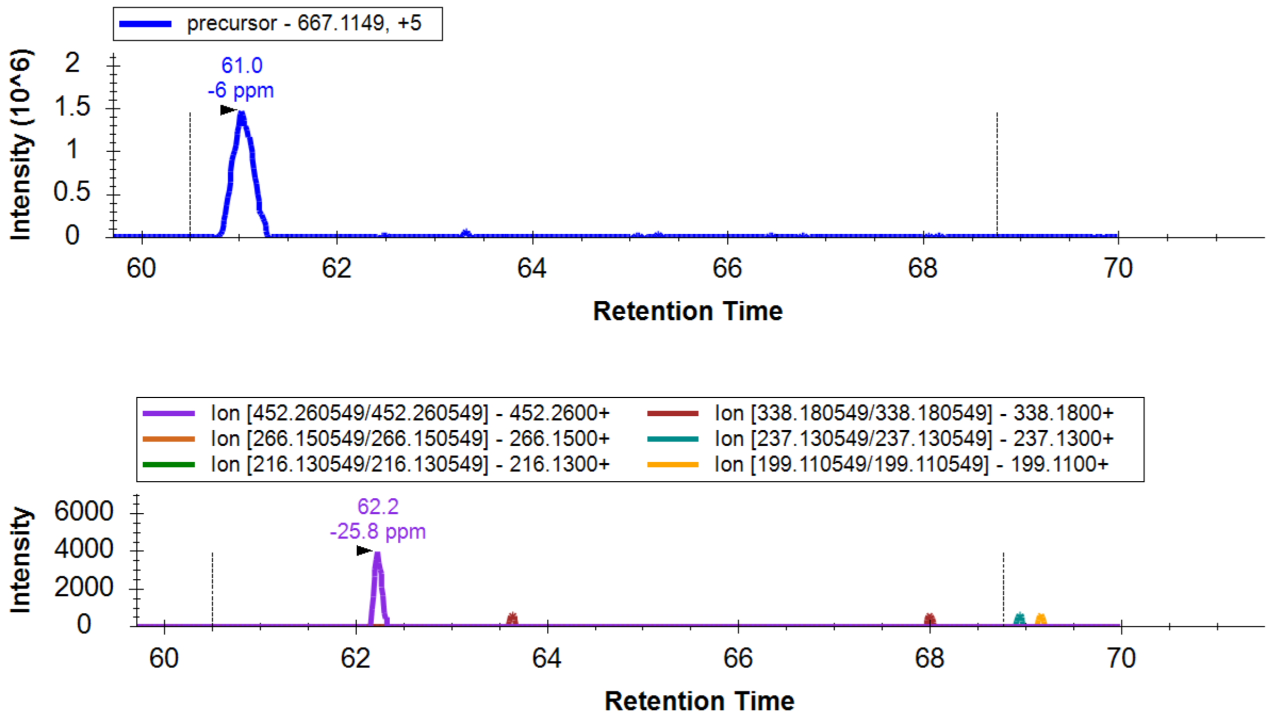

Figure S5: LC-MS/MS Analysis of nisin peptide in urine. Nisin was enriched from urine (sample 6) and analyzed by parallel reaction monitoring on a Thermo Fusion Lumos mass spectrometer. Representative extracted ion chromatograms of the intact +5 precursor ion (top) and fragment ions after collision induced dissociation of the precursor (bottom) are shown.

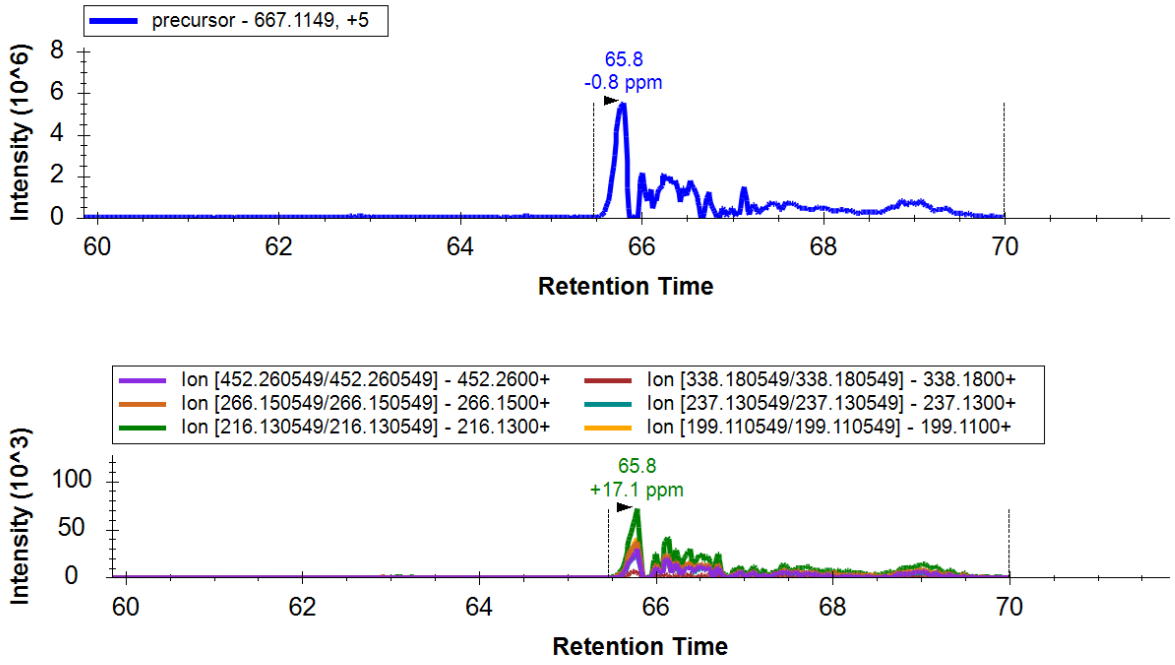

Figure S6: LC-MS/MS Analysis of nisin standards. Nisin (60 ng) was analyzed by parallel reaction monitoring on a Thermo Fusion Lumos mass spectrometer. Representative extracted ion chromatograms of the intact +5 precursor ion (top) and fragment ions after collision induced dissociation of the precursor (bottom) are shown.

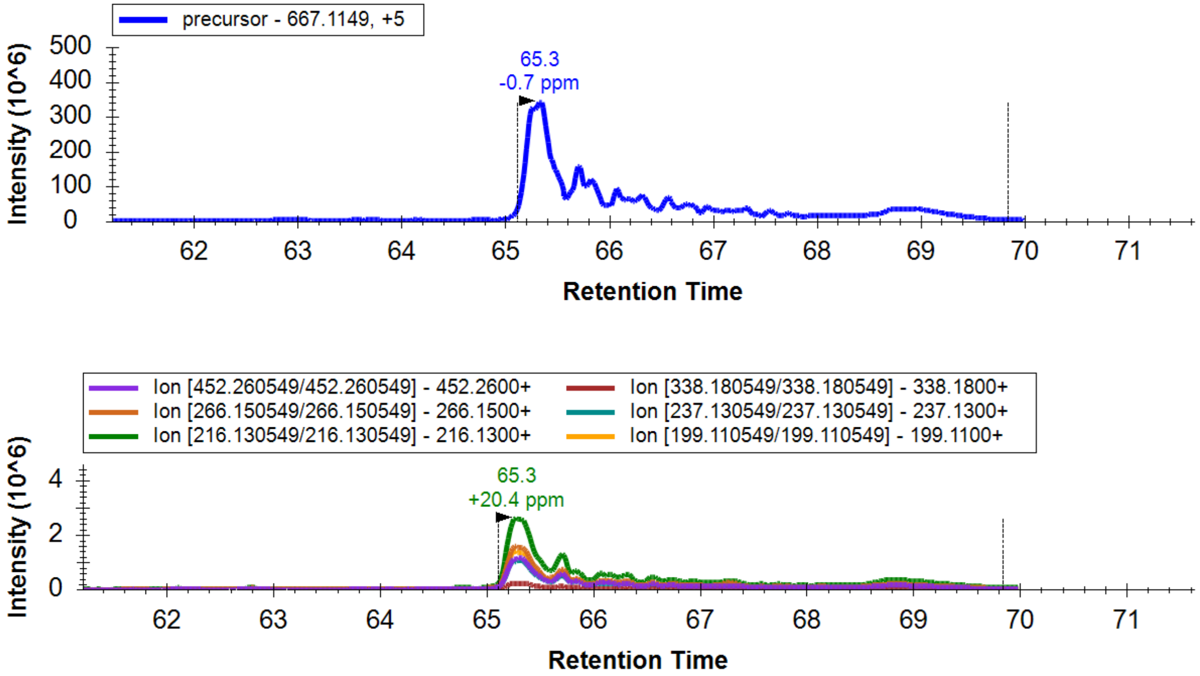

Figure S7: LC-MS/MS Analysis of nisin standards. Nisin (120 ng) was analyzed by parallel reaction monitoring on a Thermo Fusion Lumos mass spectrometer. Representative extracted ion chromatograms of the intact +5 precursor ion (top) and fragment ions after collision induced dissociation of the precursor (bottom) are shown.

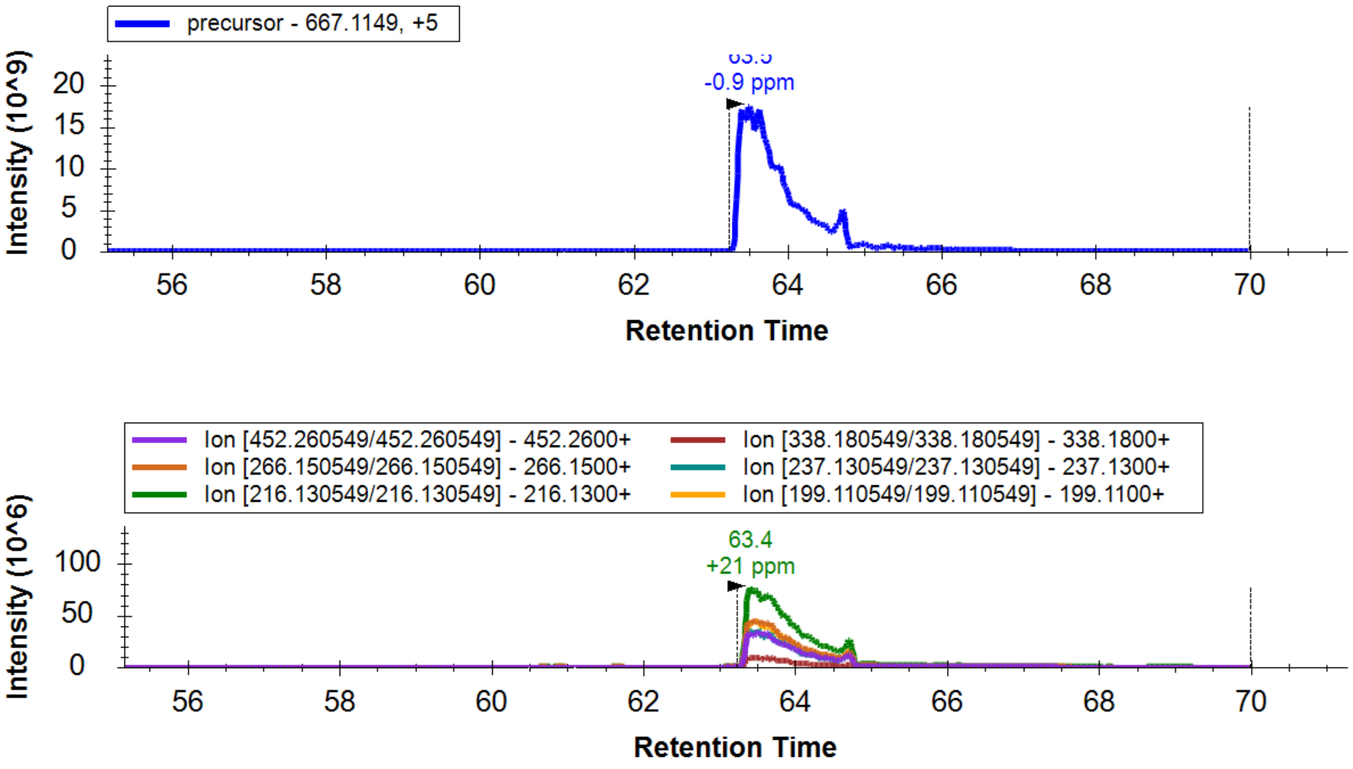

Figure S8: **LC-MS/MS Analysis of nisin standards**. Nisin (250 ng) was analyzed by parallel reaction monitoring on a Thermo Fusion Lumos mass spectrometer. Representative extracted ion chromatograms of the intact +5 precursor ion (top) and fragment ions after collision induced dissociation of the precursor (bottom) are shown.

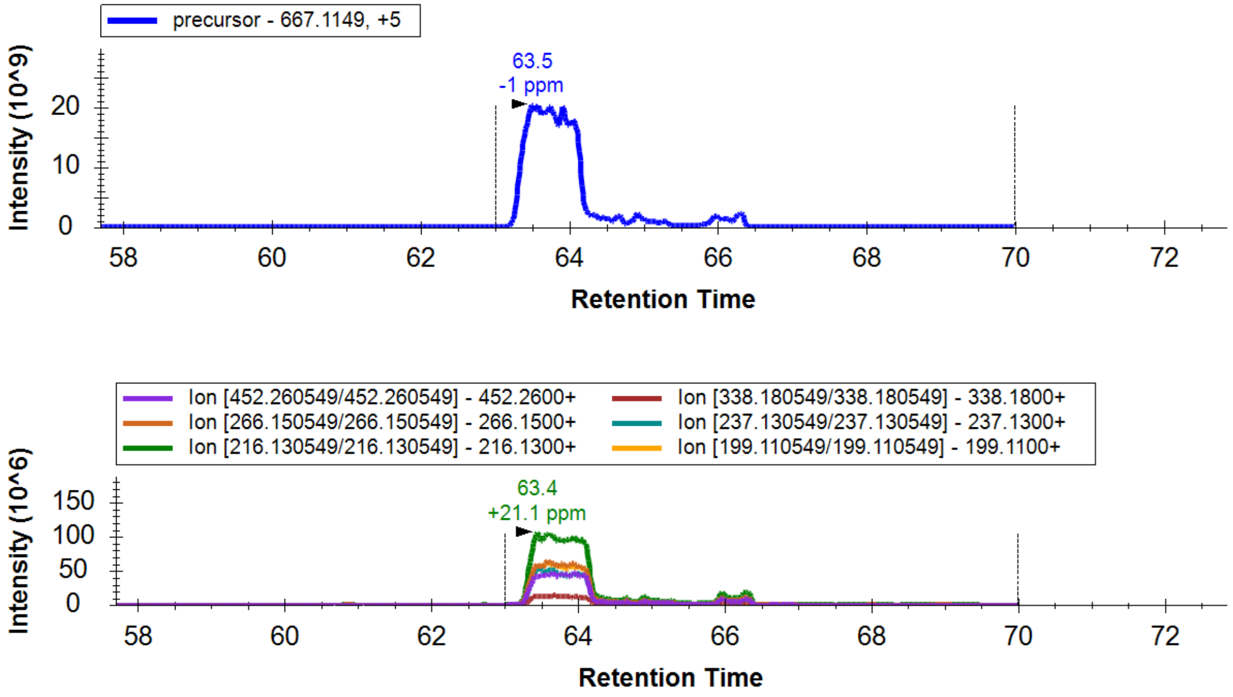

Figure S9: LC-MS/MS Analysis of nisin standards. Nisin (500 ng) was analyzed by parallel reaction monitoring on a Thermo Fusion Lumos mass spectrometer. Representative extracted ion chromatograms of the intact +5 precursor ion (top) and fragment ions after collision induced dissociation of the precursor (bottom) are shown.

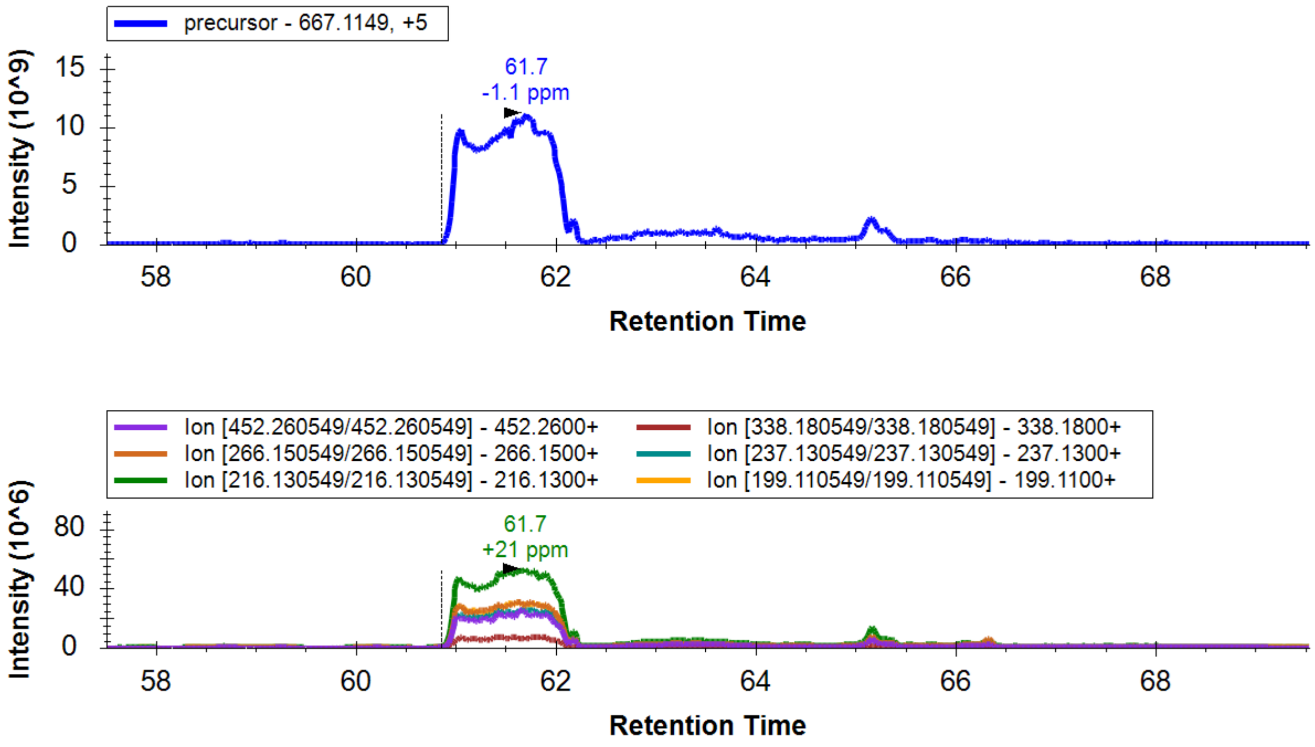

Figure S10: LC-MS/MS Analysis of nisin standards. Nisin (1000 ng) was analyzed by parallel reaction monitoring on a Thermo Fusion Lumos mass spectrometer. Representative extracted ion chromatograms of the intact +5 precursor ion (top) and fragment ions after collision induced dissociation of the precursor (bottom) are shown.
